# The Goettingen minipig as an experimental model in wound-healing studies

**DOI:** 10.1016/j.jpra.2024.03.011

**Published:** 2024-04-04

**Authors:** Dr. Wiebke Eisler, Prof. Dr. Manuel Held, Prof. Dr. Afshin Rahmanian-Schwarz, Dr. Jan-Ole Baur, Prof. Dr. Adrien Daigeler, Dr. Markus Denzinger

**Affiliations:** a Department of Hand, Plastic, Reconstructive, and Burn Surgery, BG Unfallklinik Tuebingen, Eberhard Karls University Tuebingen, Germany; b Department of Plastic, Hand, Reconstructive and Aesthetic Surgery, Hand surgery, Traunstein Hospital, Ludwig-Maximilians-Universität Munich, Germany; c Department of Dermatology, University hospital Erlangen, Friedrich-Alexander-University Erlangen-Nuernberg, Germany; d Department of Pediatric Surgery, Klinik St. Hedwig, University Medical Center Regensburg, Regensburg, Germany

**Keywords:** Animal model, Wound healing, Tissue engineering, Goettingen minipig

## Abstract

**Background:**

Deep dermal wounds in extensive burns and chronic wound-healing disorders represent a significant medical problem and require a high level of therapy to reduce the risk of infection and other long-term consequences, such as amputation. A better understanding of the wound-healing processes is essential, and animal models are indispensable to fundamental research.

**Objective:**

This study aimed to provide a transparent protocol and prove the effectiveness of an *in vivo* porcine model using Goettingen minipigs for wound-healing studies.

**Material and methods:**

Thirteen female Goettingen minipigs were kept in species-appropriate housing and were treated according to the German law for the protection of animals. The study was performed with permission from the local ethical review committee of animal welfare. The experimental procedure for studying dermal regeneration in 102 full-thickness wounds through clinical observation and histological analysis, focusing on neodermal formation, is described in detail.

**Results:**

The Goettingen minipig model proved to be suitable in wound-healing studies. The dermal regeneration was evident and viewable without wound contamination or any rejection reaction. The histological evaluations were also reliable and clearly presented the optimized wound healing of deep dermal wounds using the different therapeutic approaches.

**Conclusion:**

Given the great clinical need for alternative or complementary therapies, we considered the Goettingen minipig trial a reliable, ethically justifiable, effective, and reproducible in vivo model for wound-healing studies.

## Introduction

Deep dermal wounds of various origins require wound closure to prevent fluid and electrolyte loss, infections, metabolic imbalances, immunosuppression, pain, and amputations. Among them, extensive burn wounds and chronic wound-healing disorders represent a significant medical problem. Whereas the “gold standard” of dermal replacement is still autologous skin grafting,^1^, ^2^, ^3^ in extensive burns, the challenges include limited donor sites and donor site morbidity. In chronic wounds, normal wound-healing mechanisms can be disturbed by other underlying diseases, resulting in nonhealing wounds, such as diabetic ulcers^4^ or peripheral arterial occlusive disease.^5^ There, an open wound develops subsequent to mechanical stress due to neuropathy or lack of perfusion in the local tissue,^6^ and the complex wound-healing phases are impeded, or the relevant cell types are impaired by inhibitory influences.^7^ Such wounds have increased due to changing demographic trends and require a high level of therapy to reduce the risk of infection and other long-term consequences, such as amputation.

In this context, new therapeutic approaches for the healing of acute and chronic deep dermal wounds are needed to support the body's dermal regeneration.^8^, ^9^, ^10^ In previous studies, we evaluated different therapeutic approaches for the healing support of deep dermal wounds^11^, ^12^, ^13^, ^14^ and compared them in an *in vivo* model with Goettingen minipigs. The necessity for a better understanding of wound-healing processes makes animal models indispensable to fundamental research. The numerous published dermal regeneration and wound-healing studies, including those in pigs,^15^, ^16^, ^17^ mostly describe the results but rarely describe their method in detail.^18^, ^19^, ^20^

The purpose of the study was to provide a transparent protocol and to prove the effectiveness of the *in vivo* porcine model for wound-healing studies.

## Material and Methods

### Animals

**Figure 1:**
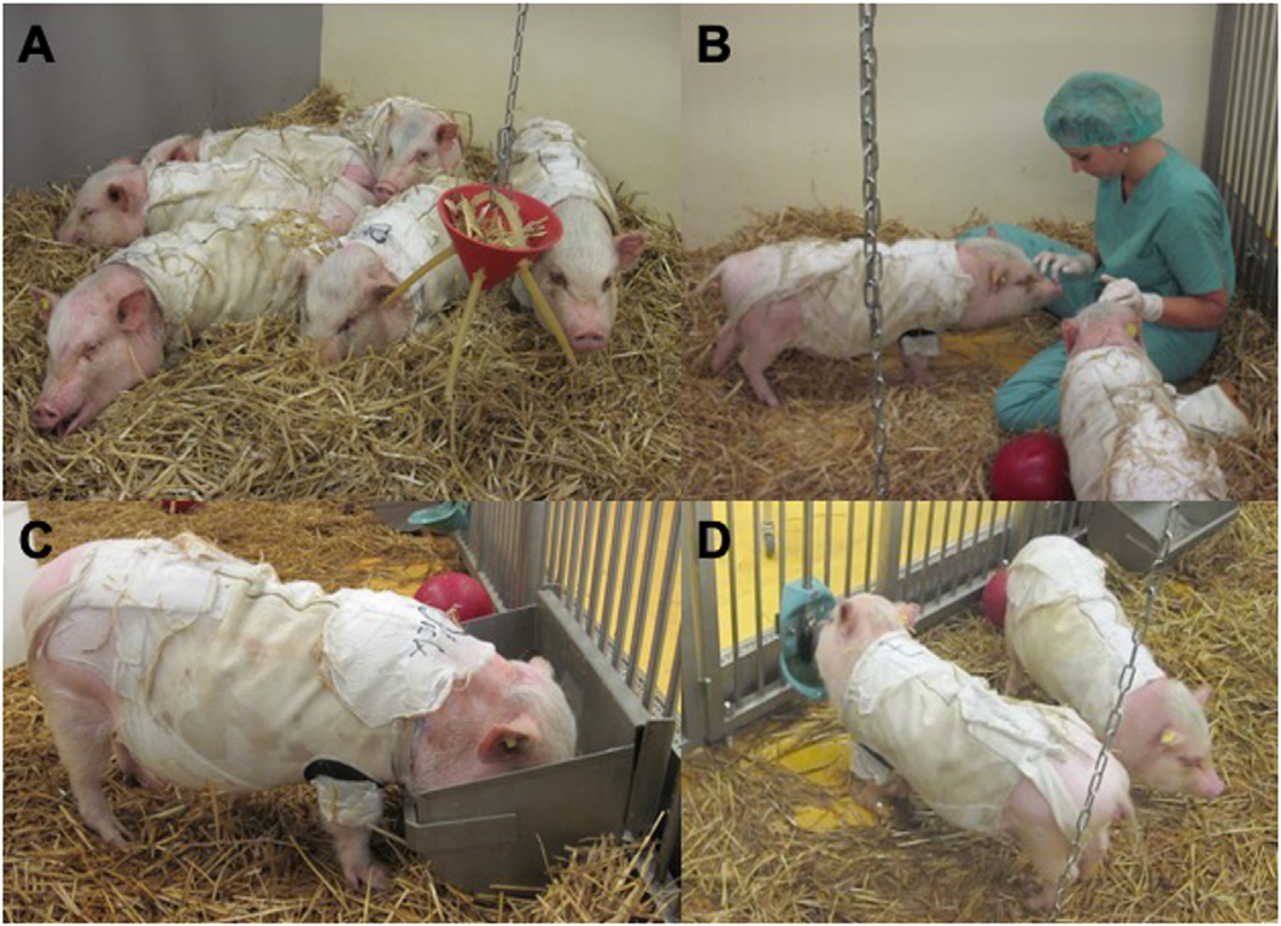
The Goettingen minipigs were housed according to the German Law on the Protection of Animals (A) in groups on straw with (B) playground equipment and entertainment by the laboratory staff, (C) troughs with a standardized minipig diet, as well as (D) water ad libitum, with a feed search enrichment function.

Animals were treated according to the German law for the protection of animals, and the study was performed with permission from the local ethical review committee for animal welfare in Baden-Wuerttemberg. Thirteen Goettingen minipigs were purchased from Ellegaard Goettingen Minipigs A/S, Dalmose, Denmark. They were all females, weighed 22.6 kg (± 1.4 kg), and aged 39 weeks (± 12 days) on arrival. The animals were kept on straw in their species-appropriate housing (a controlled pen with 21 ± 1°C, 60% relative humidity, 12 h/12 h light-dark schedule) in the large-animal laboratory of the University. They had playground equipment hanging on chains, balls, and troughs with water ad libitum, all with a feed search enrichment function (Figure 1). Every day, they were fed 400 g of a standard minipig diet (SDS SMP, Special Diets Services, Witham, Essex, UK) to ensure good health and were weighed weekly on a platform scale (PCE-PS XL, PCE Deutschland GmbH, Meschede, Germany).

### Conditioning

Prior to the first surgical procedure, the Goettingen minipigs were held for at least four weeks to acclimatize to the new environment and become accustomed to the staff (veterinarian, animal caretakers, and experimenters) and experimental conditions (Figure 1). They were conditioned by classic and operant conditioning and were trained to lie in a special pig hammock with holes for the pigs’ legs. The aim was to keep the animals calm later when treating the wound areas for bandage renewal and to avoid frequent sedation. Furthermore, they became accustomed to wearing customized swine jackets (Ellegaard Minipig Jacket Large Full Body; Lomir Biomedical Inc., Notre Dame de L'Ille Perrot, Quebec, Canada).

### Anesthesia

On day 1, the day of the dermal wounding procedure, and on day 21, for sample collection and euthanasia, the Goettingen minipigs were premedicated after a fasting period of 12h. When deep sedation occurred, they were transported to the animal surgery and fixed in a chest position on a warm mat for hypothermia prophylaxis. A peripheral intravenous catheter (16 G) was placed in one of the venous ear vessels, and anesthesia was deepened by the veterinarian, thus enabling endotracheal intubation. The animals were pressure-control ventilated via a respirator. The surgical tolerance stage was maintained, and sufficient intraoperative analgesia was initiated. The intraoperative monitoring included body temperature measurements, electrocardiography, and pulse oximetry. A blood pressure cuff was placed on the right front leg of the pig to monitor blood pressure during the experimentation. In addition, a moisturizing and protective eye ointment was applied to both eyes. Postoperative analgesia was performed using Fentanyl patches.

### Dermal wounding procedure and treatments

**Figure 2:**
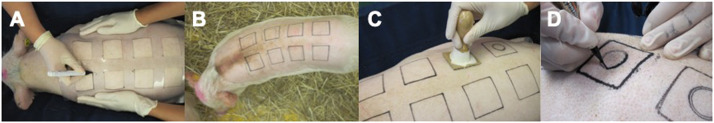
On the day of the experiment's start, the pigs’ dorsal trunks were shaved. (A) The outlines of the squares were marked with a permanent marker by using a template (B) to define standardized wound areas. (C) The circles were defined using a standardized stamp and (D) both the squares and the circles were tattooed to guarantee permanent markings.

**Figure 3:**
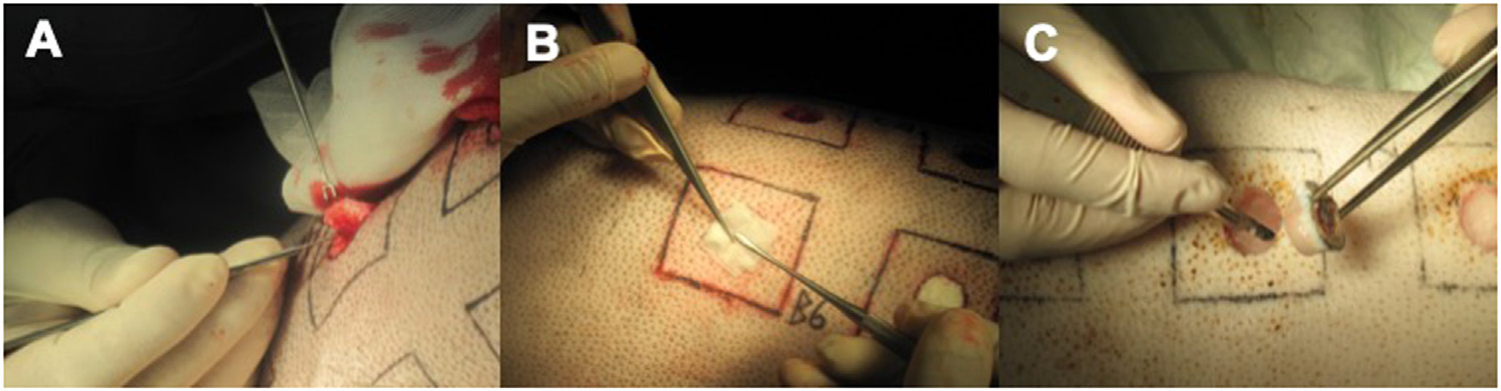
Full-thickness skin ‘defects’ with a diameter of 2.0 cm and a depth of 0.6 cm were (A) surgically created with a scalpel by excising all marked circles on each animal's back. (B) The different wound dressings were applied, and (C) after 21 days, necropsies were taken by excising the original circular wound area.

On the first day of the study, each pig's dorsal trunk between the shoulder blades and hip was shaved (Favorita II, Aesculap AG&Co.KG, Tuttlingen, Germany). A template was used to define standardized wound areas paravertebrally with a separation distance of 6.0 cm in each direction. The outlines of circles (diameter 2.0 cm) and squares (5.0 × 5.0 cm) were marked using a permanent marker and subsequently tattooed using a Professional Make-up Kit, G-9740, Giant Sun Industry Co., Ltd., New Taipei City, Taiwan (Figure 2). Sterilization of the dorsum of each minipig followed in a standardized manner. Eight full-thickness skin defects with a diameter of 2.0 cm and a depth of 0.6 cm were surgically created with a scalpel by excising all marked circles on each animal's back (Figure 3). Using a randomized controlled technique, different wound dressings and growth factors were applied topically on the day of the wounding procedure (single application) or additionally, every second day on top of the wound (multiple application) while the control wounds remained untreated:

- Collagen-gelatin matrix (animal-derived, bioresorbable, nonwoven, noncrosslinked) with various surface densities: 30 g/m^2^, 75 g/m^2^, 90 g/m^2^, and 150 g/m^2^.
- Composite biomaterial: Collagen-gelatin matrix + Growth factor (recombinant human growth and differentiation factor 5 (rhGDF-5, via expression in Escherichia coli, strain W3110 BP) at various concentrations: 100 ng/m^2^, 500 ng/m^2^, 1000 ng/m^2^, and 5000 ng/m^2^ developed in cooperation with Freudenberg New Technologies KG, Weinheim, Germany and Biopharm GmbH, Heidelberg, Germany).

In addition, two commercially available products were also applied according to the manufacturer's instructions and examined for direct comparison: a crosslinked and a noncrosslinked collagen biomatrix.

### Bandaging

Wounds were covered with sterile nonadhesive foil directly on the wounds and a sterile, airtight adhesive occlusion foil by Smith and Nephew Orthopedics (GmbH, Tuttlingen, Germany) on top to avoid fluid loss, cross-contamination, and for environmental protection to prevent bacterial contamination. The customized minipig jacket was fixed with Fixomull® tape (by BSN Medical GmbH, Hamburg, Germany) for postoperative bandage protection. Bandage renewal was carried out every second day without any necessary sedation.

### Clinical observation

**Figure 4:**
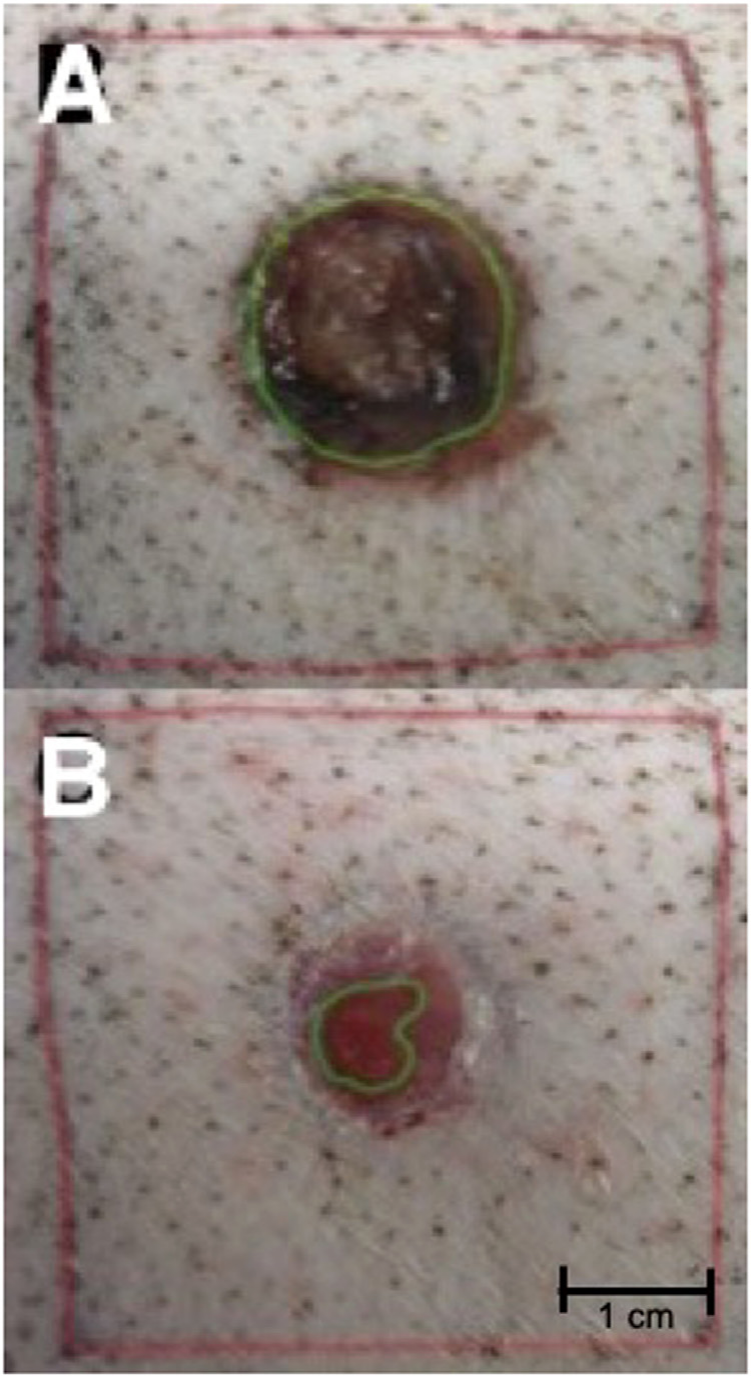
For planimetric evaluation, standardized photo documentation was performed and analyzed using Adobe Photoshop. (A) All circles and squares were outlined, so the wound area and the total area of the outer squares were calculated to (B) determine the percentage of wound closure over time during the study.

Visual wound observation and photographic documentation were performed with the bandage renewal. A customized tripod was used to identify the standardized distance to the wounds. Conventional photographs of all wounds were taken and imported into Adobe Photoshop (Adobe Systems Inc., San Jose, California, USA) for metric analysis. The wound-healing progress was analyzed planimetrically to determine the percentage of wound closure over time. In the pictures taken, all wounds and squares were outlined so the total wound area and the area of the tattooed outer square could be calculated (Figure 4).

### Histological evaluation

**Figure 5:**
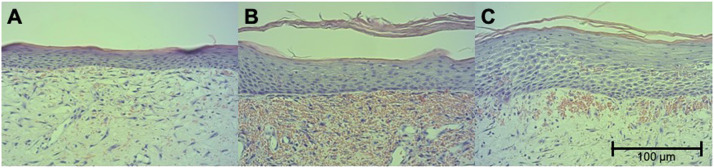
For histopathological analysis, H&E stained slides were analyzed with a particular focus on neodermal formation in (A) the untreated control group and in (B) with visible enhancements in wound regeneration in one treatment group with a marginally thicker epidermis and in (C) another treatment group with significantly thicker epidermis and higher epidermal cell density.

On the last day of the study, the minipigs were anesthetized and euthanized by the veterinarian by administering a lethal T61 (Embutramide) dose via a venous catheter. The original circular wound areas were surgically excised with a scalpel (Figure 3). The necropsies were processed for histopathology. All samples were fixed in 4% buffered paraformaldehyde for 48 hours at 4°C. Afterward, each sample was stored in phosphate-buffered saline, dehydrated in a graded ethanol bath cleared with xylene, infiltrated with paraffin wax (Shandon Pathcentre Tissue Processor, Thermo Scientific, Astmoor, Runcorn, UK), and embedded in paraffin wax blocks (Leica EG1160, Leica Biosystems Nussloch GmbH, Nussloch, Germany). The paraffin-fixed blocks were cut into 5 µm thick slides using a microtome (Leica RM2125RT, Leica Biosystems Nussloch GmbH, Nussloch, Germany), placed on slides to dry for 12 hours at 37°C, and stained with hematoxylin and eosin (H&E). For the histopathological evaluation, a Zeiss microscope (Axio Observer.Z1, Carl Zeiss Microscopy GmbH, Jena, Germany) with ZEN blue edition (2011) microscopy software (Carl Zeiss Microscopy GmbH, Jena, Germany) was used. Photographs were taken using a digital microscope camera (AxioCam ERc 5s, Carl Zeiss Microscopy GmbH, Jena, Germany). The histological analysis was performed in the former sore center of each wound with a particular focus on neoepidermal formation. The neoepidermal thickness was measured from the stratum basale to the stratum corneum of the epidermis, repeated three times at a distance of 100 µm. The cell density of epidermal cells was measured using the total number of cells within a rectangular area of 100 × 50 µm (5 mm^2^) in the neoepidermis, again in three different sections at a distance of 100 µm (Figure 5).

### Statistical analysis

The software IBM SPSS Statistics version 20.0 was used for the statistical analysis. The alpha level was set at α = 0.05. The Wilcoxon rank-sum test (Mann–Whitney U test) was performed to compare independent samples of each group.

## Results

**Figure 6:**
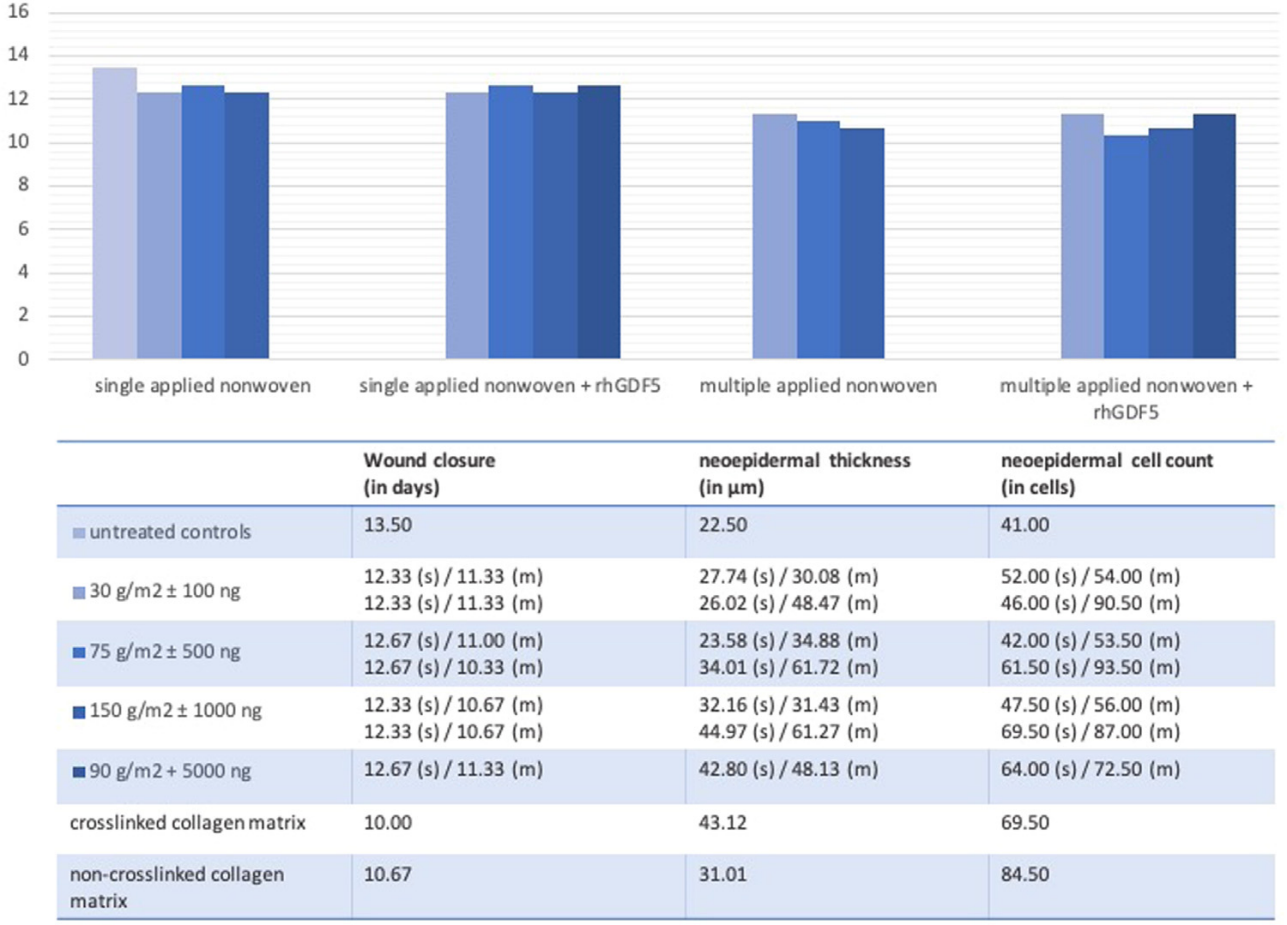
Planimetric and histological results of the single (s) and multiple (m) applied collagen-gelatin matrix with various surface densities: 30 g/m^2^, 75 g/m^2^, 90 g/m^2^,150 g/m^2^ without (1st row) or with (2nd row) the recombinant growth and differentiation factor 5 at various concentrations: 100 ng/m^2^, 500 ng/m^2^, 1000 ng/m^2^, 5000 ng/m^2^ compared to untreated controls and a commercially available crosslinked and non-crosslinked collagen biomatrix.

Within the experimental observation period of 21 days,^21^ the clinical observation and histological analysis were performed without interruptions (Figure 6). There was no wound contamination or any rejection reaction.

All wounds were covered with a continuous neoepidermis after an average of 12.44 days (single applied nonwoven: 30 g/m^2^: 12.33 [p = 0.0987]; 75 g/m^2^: 12.67 [p = 0.1775]; 150 g/m^2^: 12.33 [p = 0.0987]), 12.50 days (single applied nonwoven + rhGDF5: 30 g/m^2^ + 100 ng: 12.33 [p = 0.0987]; 75 g/m^2^ + 500 ng: 12.67 [p = 0.1775]; 150 g/m^2^ + 1000 ng: 12.33 [p = 0.0987]; 90 g/m^2^ + 5000 ng: 12.67 [p = 0.1775]), 11.00 days (multiple applied nonwoven: 30 g/m^2^: 11.33 [p = 0.0062]; 75 g/m^2^: 11.00 [p = 0.0007]; 150 g/m^2^: 10.67 [p = 0.0002]) and after 10.92 days (multiple applied nonwoven + rhGDF5: 30 g/m^2^ + 100 ng: 11.33 [p = 0.0015]; 75 g/m^2^ + 500 ng: 10.33 [p = 0.0002]; 150 g/m^2^ + 1000 ng: 10.67 [p = 0.0002]; 90 g/m^2^ + 5000 ng: 11.33 [p = 0.0021]) compared with untreated controls after 13.50 days postoperatively. The higher concentration with multiple applications, therefore, showed faster wound closure comparable with the results for the two established dermal substitutes with wound closure after 10.00 days (p < 0.0001) for the crosslinked and 10.67 days (p = 0.0002) for the noncrosslinked collagen matrix.

The mean neoepidermal thickness from the stratum basale to the stratum corneum also improved with multiple applications. Whereas the single treatment showed thinner neoepidermis (nonwoven 30 g/m^2^: 27.74 µm [p = 0.0322]; 75 g/m^2^: 23.58 µm [p = 0.7010]; 150 g/m^2^: 32.16 µm [p = 0.0329]; 30 g/m^2^ scaffold + 100 ng rhGDF-5: 26.02 µm [p = 0.0405]; 75 g/m^2^ + 500 ng: 34.01 µm [p = 0.0008]; 150 g/m^2^ + 1000 ng: 44.97 µm [p < 0.0001]; 90 g/m^2^ + 5000 ng: 42.80 µm [p < 0.0001]), with multiple applications higher values of neoepidermal thickness were reached (nonwoven 30 g/m^2^: 30.08 µm [p = 0.008]; 75 g/m^2^: 34.88 µm [p = 0.0297]; 150 g/m^2^: 31.43 µm [p = 0.0463]; 30 g/m^2^ scaffold + 100 ng rhGDF-5: 48.47 µm [p < 0.0001]; 75 g/m^2^ + 500 ng: 61.72 µm [p < 0.0001]; 150 g/m^2^ + 1000 ng: 61.27 µm [p < 0.0001]; 90 g/m^2^ + 5000 ng: 48.13 µm [p < 0.0001]) especially compared with untreated controls with a mean of 22.50 µm with higher values to the crosslinked (43.12 µm, p < 0.0001) and noncrosslinked (31.01 µm, p = 0.0015) collagen matrix.

The mean neoepidermal cell count within a section of 100 µm width [cells/5 mm^2^] was less in the single application groups (nonwoven 30 g/m^2^: 52.00 cells [p = 0.4283]; 75 g/m^2^: 42.00 cells [p = 0.3968]; 150 g/m^2^: 47.50 cells [p = 0.5774]; 30 g/m^2^ scaffold + 100 ng rhGDF-5: 46.00 cells; 75 g/m^2^ + 500 ng: 61.50 cells; 150 g/m^2^ + 1000 ng: 69.50 cells [p = 0.0132]; 90 g/m^2^ + 5000 ng: 64.00 cells [p = 0.0229]) than in the multiple application groups (nonwoven 30 g/m^2^: 54.00 cells [p = 0.1269]; 75 g/m^2^: 53.50 cells [p = 0.1167]; 150 g/m^2^: 56.00 cells [p = 0.1307]; 30 g/m^2^ scaffold + 100 ng rhGDF-5: 90.50 cells [p = 0.0118]; 75 g/m^2^ + 500 ng: 93.50 cells [p = 0.0174]; 150 g/m^2^ + 1000 ng: 87.00 cells [p = 0.0019]; 90 g/m^2^ + 5000 ng: 72.50 cells [p = 0.0374]) and in total higher than in untreated controls with a mean of 41.00 cells but did not reach values like the crosslinked (69.50 cells, p = 0.0089) and noncrosslinked (84.50 cells, p = 0.0002) collagen matrix.

Blood screening tests taken from each animal showed no systemic effects. Termination criteria like therapy-resistant infectious wounds, systemic effects, therapy-resistant pain conditions, no water/ feed intake, and abnormal behaviors such as panic could not be observed.

## Discussion

Certainly, there are reservations about animal experiments. However, the development of medical treatments requires adequate experimental techniques, at least when it comes to the approval of products and their use in humans. Testing always starts at the molecular level in cell cultures,^22^ but to estimate the effect in humans without harming humans, *in vivo* studies are indispensable^19^ before a product can be tested in a clinical situation.^23^ At present, there are no alternative methods for this field of research. No artificial skin models or cell culture approaches can simulate the situation of a native wound with all its skin layers or come close to what can be gained through in vivo knowledge. There are numerous commercially available human skin equivalents on the market for specific experimental purposes, e.g., Epiderm^TM^ by MatTek (neonatal human-derived epidermal keratinocytes). Basically, these are differentiated into pure keratinocyte models (monolayer cultures or fully differentiated models) for epidermal research and into skin equivalent models with dermal components like Episkin^TM^ by SkinEthic (human keratinocytes cultured on a collagen base). The use of skin models has significantly reduced the number of animal studies used for skin compatibility in pharmaceuticals and cosmetics.^24^ However, every skin model excludes the natural variations that occur in humans. Each model is limited in its possibilities and cannot represent such a complex process as wound healing.^4^ This applies in particular to the migration of keratinocytes, the regeneration from the dermis, the involvement of natural endogenous growth factors, vitamins, salts, etc., the revascularization of wound tissue (angiogenesis), and immunological and inflammatory reactions. To evaluate the wound-healing reactions of deep dermal wounds in vivo, it is necessary to study them in a standardized animal model. Therefore, the specific choice of animal model for each *in vivo* study is important, because every animal model has its unique advantages and disadvantages.

In this respect, Goettingen minipigs represent an effective wound model^25^^,^^26^ as porcine skin provides recognizable similarities to human skin in anatomical structure^27^^,^^28^ and its biochemical and physiological metabolic properties. The skin architecture^15^ and physiology, including the general phases of wound healing (inflammation, proliferation, and maturation), of the human and minipig are alike.^23^ The epidermis and dermis reveal structural similarities,^29^ and the arrangement and distribution of the connective tissue elements are very similar. Even the collagen structure, as the main component of the skin, is almost identical in porcine and human skin. Another advantage of the pig as a skin model is its high similarity in vascularization to human skin.^30^ Already published studies show that wound contraction is significantly lower in a pig model than in a rat model. The advantages of the minipigs compared with domestic pigs are that the minipigs are small, easy to handle, and, unlike the domestic pig, genetically and phenotypically controlled,^29^ which can be ascribed to the standardized conditions under which Göttingen minipigs are raised. Of particular interest is the nonpigmented skin as it eases wound observation. The disadvantage is the high cost (in general, for acquisition and laboratory fees), which corresponds to the disadvantages of most large-animal models. Nevertheless, the pig model could not be replaced by a species with physiologically lower senses due to its expressiveness and with regard to the questions the study asks. The clinical problem is mainly directed at deep dermal wounds in burns and chronic wounds, especially in people with diabetes. The planned *in vivo* tests were nevertheless performed on freshly set wounds in healthy animals. If increased wound infection or superinfection would have been found in the healthy animals, this might have caused severe complications in diabetic animals. The general effects must first be tested under physiological conditions to define the reference situation for basic research. Because of this, complex and stressful diabetes model situations should follow later.

Many articles in the literature describe the use of pigs in studies.^23^^,^^29^^,^^31^, ^32^, ^33^, ^34^, ^35^, ^36^ Nevertheless, there is a lack of detailed protocols in the literature. In the present study, the experimental setup with Goettingen minipigs has proven reliable and representative of dermal wound healing in a low-stress experimental environment. Standardized wounds can be generated to study wound-healing disorders and treatments with high clinical relevance. On the other hand, we did observe wound contraction in our study. For this reason, wound healing from the wound bed rather than the wound edges could not be asserted with absolute certainty. However, the evaluation tools for dermal regeneration, in terms of clinical observation and histological analysis with a particular focus on neodermal formation, were effective and practicable without any inconvenience. Considering this, we have to thank our intensive daily animal training and conditioning prior to the study for the good conditions experienced during the study. The acclimatization and conditioning of the pigs led to a drastic reduction in the stress potential for all participants. Therefore, anesthetics and sedation could be limited to the minimum and were only used for the surgical procedures on day 1 and day 21, without distressing the animals. Overall, the initial investment of time and patience in the conditioning contributed significantly to the low-stress execution of the experiment and the success of the study.

## Conclusion

Considering the great clinical need for alternative or complementary forms of therapy for deep dermal wound healing, we considered the Goettingen minipig trial to be a reliable, ethically justifiable, effective, and reproducible *in vivo* model for future wound-healing studies.

## Ethical Approval Statement

The study was performed with permission from the local ethical review committee for animal welfare in Baden-Wuerttemberg (AT 1/12).

## CRediT authorship contribution statement

**Dr. Wiebke Eisler:** Conceptualization, Methodology, Validation, Investigation, Data curation, Writing – original draft, Writing – review & editing. **Prof. Dr. Manuel Held:** Conceptualization, Methodology, Software, Validation, Investigation, Data curation, Visualization. **Prof. Dr. Afshin Rahmanian-Schwarz:** Conceptualization, Methodology, Resources, Supervision, Project administration. **Dr. Jan-Ole Baur:** Software, Formal analysis, Validation, Investigation, Data curation. **Prof. Dr. Adrien Daigeler:** Resources, Supervision, Project administration. **Dr. Markus Denzinger:** Formal analysis, Writing – original draft, Visualization.

## Declaration of competing Interest

None declared.

## References

[1] Andreassi A. (2005). Classification and pathophysiology of skin grafts. Clinics in dermatology.

[2] Stanton R.A., Billmire D.A. (2002). Skin resurfacing for the burned patient. Clinics in plastic surgery.

[3] Supp D.M., Boyce S.T. (2005). Engineered skin substitutes: practices and potentials. Clinics in dermatology.

[4] Papanas N., Maltezos E. (2007). Growth factors in the treatment of diabetic foot ulcers: new technologies, any promises?. Int J Low Extrem Wounds.

[5] Eneroth M., Persson B.M. (1993). Risk factors for failed healing in amputation for vascular disease. A prospective, consecutive study of 177 cases. Acta Orthop Scand.

[6] Vogt P.M. (2007). [Innovative wound therapy and skin substitutes for burns]. Chirurg.

[7] Harding K.G., Morris H.L., Patel G.K. (2002). Science, medicine and the future: healing chronic wounds. BMJ.

[8] Auger F.A. (2004). Tissue-engineered skin substitutes: from in vitro constructs to in vivo applications. Biotechnol Appl Biochem.

[9] Dieckmann C. (2010). Regenerative medicine in dermatology: biomaterials, tissue engineering, stem cells, gene transfer and beyond. Exp Dermatol.

[10] van der Veen V.C. (2010). Biological background of dermal substitutes. Burns.

[11] Held M. (2016). A novel collagen-gelatin scaffold for the treatment of deep dermal wounds-an evaluation in a minipig model. Dermatol Surg.

[12] Petersen W. (2016). The use of collagen-based matrices in the treatment of full-thickness wounds. Burns.

[13] Schiefer J.L. (2017). Growth differentiation factor 5 accelerates wound closure and improves skin quality during repair of full-thickness skin defects. Adv Skin Wound Care.

[14] Schiefer J.L. (2016). Frequent application of the new gelatin-collagen nonwoven accelerates wound healing. Adv Skin Wound Care.

[15] Avon S.L., Wood R.E. (2005). Porcine skin as an in-vivo model for ageing of human bite marks. J Forensic Odontostomatol.

[16] Jung Y. (2013). Experimental pig model of clinically relevant wound healing delay by intrinsic factors. Int Wound J.

[17] Rothenberger J. (2014). Development of an animal frostbite injury model using the Goettingen-Minipig. Burns.

[18] Michael S. (2013). Tissue engineered skin substitutes created by laser-assisted bioprinting form skin-like structures in the dorsal skin fold chamber in mice. PLoS One.

[19] Middelkoop E. (2004). Porcine wound models for skin substitution and burn treatment. Biomaterials.

[20] Ploemen I.H. (2014). Minipigs as an animal model for dermal vaccine delivery. Comp Med.

[21] Fu X. (2007). Adipose tissue extract enhances skin wound healing. Wound Repair Regen.

[22] Naves L.B. (2016). In vitro skin models and tissue engineering protocols for skin graft applications. Essays Biochem.

[23] Glerup P. (2011). The Minipig in Biomedical Research.

[24] Zhang Z., Michniak-Kohn B.B. (2012). Tissue engineered human skin equivalents. Pharmaceutics.

[25] Stricker-Krongrad A., Shoemake C.R., Bouchard G.F. (2016). The miniature swine as a model in experimental and translational medicine. Toxicol Pathol.

[26] Summerfield A., Meurens F., Ricklin M.E. (2015). The immunology of the porcine skin and its value as a model for human skin. Mol Immunol.

[27] Qvist M.H. (2000). Evaluation of Gottingen minipig skin for transdermal in vitro permeation studies. Eur J Pharm Sci.

[28] Sullivan T.P. (2001). The pig as a model for human wound healing. Wound Repair Regen.

[29] Qvist M.H. (2000). Evaluation of Gottingen minipig skin for transdermal in vitro permeation studies. European journal of pharmaceutical sciences: official journal of the European Federation for Pharmaceutical Sciences.

[30] Hunziker E.B. (2002). Articular cartilage repair: basic science and clinical progress. A review of the current status and prospects. Osteoarthritis Cartilage.

[31] Becker S.T. (2010). Comparison of vacuum and conventional wound dressings for full thickness skin grafts in the minipig model. International journal of oral and maxillofacial surgery.

[32] Dame M.K. (2008). Establishment and characteristics of Gottingen minipig skin in organ culture and monolayer cell culture: relevance to drug safety testing. In vitro cellular & developmental biology. Animal.

[33] Danks W.H.a.A. (2010).

[34] Fu X. (2007). Adipose tissue extract enhances skin wound healing. Wound repair and regeneration: official publication of the Wound Healing Society [and] the European Tissue Repair Society.

[35] Van Dorp A.G. (1998). Dermal regeneration in full-thickness wounds in Yucatan miniature pigs using a biodegradable copolymer. Wound repair and regeneration: official publication of the Wound Healing Society [and] the European Tissue Repair Society.

[36] Reagan B.J. (1997). Analysis of cellular and decellular allogeneic dermal grafts for the treatment of full-thickness wounds in a porcine model. The Journal of trauma.

